# Parental and infant characteristics and childhood leukemia in Minnesota

**DOI:** 10.1186/1471-2431-8-7

**Published:** 2008-02-25

**Authors:** Kimberly J Johnson, John T Soler, Susan E Puumala, Julie A Ross, Logan G Spector

**Affiliations:** 1 Division of Epidemiology/Clinical Research, Department of Pediatrics, University of Minnesota, Minneapolis, Minnesota, USA; 2 Minnesota Cancer Surveillance System, Minnesota Department of Health, St. Paul, Minnesota, USA; 3 University of Minnesota Cancer Center, Minneapolis, Minnesota, USA

## Abstract

**Background:**

Leukemia is the most common childhood cancer. With the exception of Down syndrome, prenatal radiation exposure, and higher birth weight, particularly for acute lymphoid leukemia (ALL), few risk factors have been firmly established. Translocations present in neonatal blood spots and the young age peak of diagnosis suggest that early-life factors are involved in childhood leukemia etiology.

**Methods:**

We investigated the association between birth characteristics and childhood leukemia through linkage of the Minnesota birth and cancer registries using a case-cohort study design. Cases included 560 children with ALL and 87 with acute myeloid leukemia (AML) diagnoses from 28 days to 14 years. The comparison group was comprised of 8,750 individuals selected through random sampling of the birth cohort from 1976–2004. Cox proportional hazards regression specific for case-cohort studies was used to compute hazard ratios (HR) and 95% confidence intervals (CIs).

**Results:**

Male sex (HR = 1.41, 95% CI 1.16–1.70), white race (HR = 2.32, 95% CI 1.13–4.76), and maternal birth interval ≥ 3 years (HR = 1.31, 95% CI 1.01–1.70) increased ALL risk, while maternal age increased AML risk (HR = 1.21/5 year age increase, 95% CI 1.0–1.47). Higher birth weights (>3798 grams) (HRALL = 1.46, 1.08–1.98; HRAML = 1.97, 95% CI 1.07–3.65), and one minute Apgar scores ≤ 7 (HRALL = 1.30, 95% CI 1.05–1.61; HRAML = 1.62, 95% CI 1.01–2.60) increased risk for both types of leukemia. Sex was not a significant modifier of the association between ALL and other covariates, with the exception of maternal education.

**Conclusion:**

We confirmed known risk factors for ALL: male sex, high birth weight, and white race. We have also provided data that supports an increased risk for AML following higher birth weights, and demonstrated an association with low Apgar scores.

## Background

Leukemia is the most common cancer occurring in children under age 15 in the United States with an annual incidence of 45.5 cases per million [1]. Most cases occur in children less than 5 years with a peak incidence at 2–3 years [2]. Translocations commonly found in childhood leukemia have been detected in neonatal blood spots, in some cases more than 10 years before the onset of disease [3]. The early age at which leukemia diagnoses occur together with the presence of leukemic translocations detected at birth implicates a role for prenatal exposures in childhood leukemia etiology.

Birth characteristics and risk of childhood leukemia have been the focus of many studies and have been addressed in several reviews [2,4,5]. Variables previously examined include parental sociodemographic characteristics, maternal reproductive history, pregnancy conditions, labor and delivery factors, and infant characteristics. Despite decades of research, few definitive risk factors have been identified. The weight of the evidence to date indicates that prenatal exposure to diagnostic radiation, certain genetic syndromes (particularly Down syndrome), male sex, and high birth weight occur more frequently in children subsequently diagnosed with leukemia [2,5].

The objective of this study was to examine the relation between characteristics recorded in birth records and childhood leukemia using a case-cohort study design which is relatively resistant to recall and selection biases that are of concern in case-control studies of childhood leukemia that frequently use survey based data collection methods.

## Methods

### Study population

All childhood cancer cases diagnosed from 1988–2004 between the ages of 28 days and 14 years were ascertained from the Minnesota Cancer Surveillance System (MCSS) and linked to their birth records through probabilistic record linkage [6]. It is estimated that MCSS ascertains 99.9% of cancer cases [7]. A case-cohort study design was implemented, for which a comparison group was selected without regard to case status and is referred to as the sub-cohort [8]. In particular, four children per cancer case were randomly selected from births recorded among Minnesota residents during 1976–2004, matching on birth year. To maintain consistent exclusion criteria between cases and the sub-cohort, subjects who died during the neonatal period who were born in 1980 (the earliest year neonatal deaths were recorded) or later were excluded. For the present analysis, childhood cancer cases other than leukemia were excluded, resulting in more than four sub-cohort children matched to each case. One subject with a diagnosis of AML following lymphoma was excluded due to the possibility that the AML was therapy related. Individuals with reported Down syndrome (n = 14) were also excluded from all analyses except where noted. The University of Minnesota Institutional Review Board (IRB), the Minnesota Department of Health (MDH) IRB, and the MCSS approved all protocols for data use.

Childhood leukemia case groups were defined according to the International Classification of Childhood Cancer (ICCC) code [9] and included: 1) acute lymphoblastic leukemia (ALL), 2) acute myeloid leukemia (AML), 3) chronic myeloproliferative disease, 4) myelodysplastic syndrome and other myeloproliferative disease and 5) unspecified and reticuloendothelial leukemias.

### Variables

Birth certificate variables that were examined included: parental sociodemographic characteristics: parental age, parental race, maternal birthplace, education, marital status; maternal reproductive characteristics: plurality, previous live births, prior fetal loss, last fetal death and live birth intervals; index pregnancy conditions: anemia, diabetes (none, pre-existing or gestational), hypertension (none, chronic, pregnancy-associated or eclampsia), adequacy of prenatal care index [10]; labor and delivery characteristics: labor induction, delivery method; and infant characteristics: sex, birth weight categorized by percentile and as a linear term, size for gestational age, gestational age in weeks, one and five minute Apgar score, assisted ventilation, Down syndrome, and congenital abnormalities. Two gestational age variables were available in birth records: 1) imputed from last menstrual period and 2) the physician's estimate. The physician's estimate of gestational age was used where the imputed gestational age was missing (n = 644 (7.3%)). Size for gestational age was calculated using the derived gestational age variable according to the method of Brenner et al [11].

### Statistical analysis

SAS version 9.1 (SAS Institute, Cary, NC) was used for all statistical analyses. A modification of the stratified Cox proportional hazards model was used to compute risk estimates using a macro written by Langholz and Jiao [8] that computes standard errors using an asymptotic variance estimator appropriate for the analysis of case-cohort designs. All models were stratified by birth year. Person-years of follow-up was calculated as the time from birth to cancer diagnosis date, the age the child turned 15, or the age of the subject on 12/31/2004, whichever came first with modification of follow-up time for cases who were also selected in the sub-cohort (n = 3) [8].

Variables were selected for multivariate models using a likelihood-ratio (LR) chi-square p-value cut-off of 0.1 for ALL comparing the intercept only model to that containing the covariate. For AML, only results from birth year stratified models are reported due to the small number of AML cases. Heterogeneity in ALL risk factors by sex or age group (<5 vs. 5–14 years) were examined by including an interaction term between sex or age group and the variable of interest. Tests of statistical significance for heterogeneity of risk were conducted using the test statement in PROC PHREG using a statistical criterion of p < 0.05.

## Results

A total of 831 cases of leukemia occurring in children diagnosed from 28 days through 14 years were ascertained by MCSS from 1988–2004. Of these, 695 (84%) were matched to birth records including 12 leukemia cases with Down syndrome (Table 1). Linkage success decreased by age at diagnosis with 88.9%, 84.1%, and 68.4% of cases matching in the 0–4, 5–9, and 10–14 year old age groups respectively. ALL diagnoses were most frequent (81%), followed by AML (14%), chronic myeloproliferative disease (1.7%), myelodysplastic syndrome and other myeloproliferative diseases (1.0%), and unspecified and reticuloendothelial leukemias (2.6%). The majority of cases occurred in children under age 5 years for both ALL and AML (Table 1). Due to the small number of cases with other leukemia types, further analyses were limited to ALL and AML cases.

**Table 1:** Distribution of leukemia cases diagnosed in 0–14 year olds in Minnesota from 1988–2004.

	**Age group**			
	**0–4 years**	**5–9 years**	**10–14 years**	**Total**

Acute lymphoblastic leukemia*	320 (56%)	163(29%)	79 (14%)	**562(81%)**
Acute myeloid leukemia^+^	55 (57%)	20 (21%)	21 (22%)	**96 (14%)**
Chronic myeloproliferative disease	6 (50%)	3 (25%)	3 (25%)	**12 (1.7%)**
Myelodysplastic syndrome and other myeloproliferative diseases	4 (57%)	2 (29%)	1 (14%)	**7 (1.0%)**
Unspecified and reticuloendothelial leukemias	6 (33%)	8 (44%)	4 (22%)	**18 (2.6%)**

**Total**	**391 (56%)**	**196 (28%)**	**108 (16%)**	**695**

^*includes 2 individuals with Down syndrome^

^+^^includes 10 individuals with Down syndrome^

Table 2 reports birth year adjusted results of regression modeling for the association between parental characteristics and childhood leukemia. Maternal education greater than high school increased the risk for ALL (HR = 1.21, 95% CI, 1.0–1.47), while maternal report of black compared to white race in birth records decreased the risk by approximately 60%. Factors associated with an increased risk for AML were maternal age (HR = 1.21 per 5 year increase, 95% CI, 1.0–1.47), and maternal foreign birthplace (HR = 2.18, 95% CI, 1.14–4.18). No particular national origin was overrepresented; three cases each had mothers who were reported to have been born in Laos and Canada, while one case mother each was born in Germany, El Salvador, Korea, Nigeria, and Peru. Similar associations were found for ALL for paternal age and race and for AML for paternal age. No other significant associations were found for paternal characteristics: race (AML), or education (ALL and AML) (data not shown).

**Table 2:** Birth year adjusted analyses of parental sociodemographic characteristics and childhood leukemia.

	**Sub-cohort**	**ALL**			**AML**		
	**No. (%)**	**No. (%)**	**HR**	**95% CI**	**No. (%)**	**HR**	**95% CI**

**Maternal age (continuous/5 year increase)**	8750	559	1.05	0.97–1.14	87	1.21	1.00–1.47
missing	0	1			0		
**Maternal Race**							
black	324 (4)	9 (2)	0.41	0.21–0.79	4 (5)	1.20	0.43–3.33
other/unknown	519 (6)	30 (5)	0.87	0.60–1.28	6 (7)	1.16	0.50–2.68
white	7907 (90)	520 (93)	ref.		77 (89)	ref.	
missing	0 (0)	1 (0)			0 (0)		
**Maternal birthplace**							
U.S. or U.S. territory	8226 (94)	532 (95)	ref.		76 (87)	ref.	
foreign born	516 (6)	26 (5)	0.76	0.51–1.15	11 (13)	2.18	1.14–4.18
missing	8 (0)	2 (0)			0 (0)		
**Maternal Education**							
high school	3173 (38)	179 (34)	ref.		29 (36)	ref.	
<high school	902 (11)	49 (9)	0.96	0.69–1.33	8 (10)	0.97	0.44–2.14
>high school	4209 (51)	301 (57)	1.21	1.00–1.47	44 (54)	1.13	0.70–1.81
missing	466 (5)	31 (6)			6 (7)		
**Marital status**							
married	6988 (80)	459 (82)	ref.		75 (86)	ref.	
not married	1754 (20)	100 (18)	0.84	0.67–1.05	12 (14)	0.61	0.33–1.13
missing	8 (0)	1 (0)			0 (0)		

Regression results for maternal reproductive, pregnancy and labor and delivery characteristics are reported in Table 3. Children whose mothers had a last fetal death interval of <3 years compared to those whose mothers had no fetal deaths were at an increased risk for ALL (HR = 1.30, 95% CI, 1.00–1.69). Children who were 3 or more years younger than their previous live born sibling compared to those who had no live siblings were also at an increased risk for ALL (HR = 1.40, 95% CI, 1.12–1.75). No other notable differences were found for maternal reproductive variables. We did not find evidence to suggest differences in pregnancy history or labor and delivery characteristics between cases and the sub-cohort with the exception of a marginally significant decreased risk for AML associated with less than adequate prenatal care (HR = 0.57, 95% CI 0.33–1.0) and an increased AML risk associated with maternal gestational weight gain of 30 or more pounds (HR = 2.59, 95% CI 1.27–5.31) based on few cases. Of note is that the association between maternal gestational weight gain and AML remained after adjustment for birth weight (data not shown).

**Table 3:** Birth year adjusted analyses of maternal reproductive characteristics, pregnancy history, and labor and delivery variables and childhood leukemia.

	**Sub-cohort**	**ALL**			**AML**		
	**No. (%)**	**No. (%)**	**HR**	**95% CI**	**No. (%)**	**HR**	**95% CI**

**Plurality**							
1	8526 (97)	547 (98)	ref.		86 (99)	ND**	
≥2	224 (3)	12 (2)	0.82	0.46–1.48	1 (1)		
missing	1 (0)	1 (0)			0 (0)		
**Previous live births**							
0	3449 (40)	202 (37)	ref.		29 (33)	ref.
1–2	4251 (49)	286 (52)	1.15	0.95–1.38	49 (56)	1.39	0.87–2.20
≥3	903 (11)	61 (11)	1.14	0.85–1.54	9 (10)	1.20	0.56–2.54
missing	147 (2)	11 (2)			0 (0)		
**Prior fetal loss**							
any	1866 (22)	134 (25)	1.14	0.94–1.41	19 (22)	1.00	0.59–1.68
none	6711 (78)	407 (75)	ref.		68 (78)		ref.
missing	173 (3)	19 (3)			0 (0)		
**Last fetal death interval**							
no fetal deaths	6564 (80)	404 (77)	ref.		67 (82)		ref.
<3 years	891 (11)	73 (14)	1.30	1.00–1.69	8 (10)	0.92	0.44–1.93
≥3 years	722 (9)	51 (10)	1.13	0.83–1.53	7 (8)	0.96	0.44–2.11
missing	573 (7)	32 (6)			5 (6)		
**Last live birth interval**							
no live births	3449 (40)	202 (37)	ref.		29 (35)	ref.
<3 years	3333 (39)	200 (36)	1.02	0.84–1.25	34 (40)	1.22	0.74–2.02
≥3 years	1786 (21)	148 (27)	1.40	1.12–1.75	21 (25)	1.40	0.79–2.47
missing	182 (2)	10 (2)			3 (3)		
Maternal diabetes*							
any	110 (3)	8 (3)	1.00	0.48–2.08	0 (0)		ND
none	3194 (97)	229 (97)	ref.		33 (100)		
missing	215 (6)	9 (4)					
**Gestational anemia**							
yes	80 (2)	8 (3)	1.52	0.72–3.19	1 (2)		ND
no	4612 (98)	305 (97)	ref.		42 (98)		
missing	291 (6)	23 (7)			4 (9)		
**Maternal hypertension**							
any	337 (4)	28 (6)	1.32	0.88–1.96	5 (6)	1.40	0.56–3.49
none	7678 (96)	481 (94)	ref.		76 (94)		ref.
missing	735 (8)	51 (9)			6 (7)		
**Intrauterine procedures**							
yes	203 (2)	13 (2)	1.01	0.57–1.78	2 (2)	0.97	0.24–4.00
no	8181 (98)	515 (92)	ref.		81 (98)		ref.
missing	366 (4)	32 (6)			4 (5)		
**Maternal weight gain**^+^							
< 30 lbs	2323 (60)	148 (56)	ref.		12 (36)		ref.
≥ 30 lbs	1526 (40)	115 (44)	1.17	0.91–1.52	21 (64)	2.59	1.27–5.31
missing	1134 (23)	73 (22)			14 (30)		
**Prenatal care index**^†^							
less than adequate	2300 (33)	146 (31)	0.95	0.77–1.16	16 (22)	0.57	0.33–1.00
adequate	4747 (67)	323 (69)	ref.		57 (78)		ref.
missing	1151 (14)	72 (13)			11 (13)		
**Induction of labor**^‡^							
yes	1432 (27)	92 (27)	0.97	0.75–1.24	18 (39)	1.67	0.91–3.04
no	3878 (73)	244 (73)	ref.		28 (61)		ref.
missing	225 (4)	19 (5)			4 (8)		
**Type of delivery**							
vaginal	6952 82)	432 (82)	ref.		73 (86)		ref.
caesarean section	1485 (18)	98 (18)	1.03	0.82–1.30	12 (14)	0.75	0.41–1.39
missing	313 (4)	30 (5)			2 (2)		

*collected 1992–2004.

^+^collected 1989–2004.

^†^collected 1980–2004.

^‡^collected 1976–1979, 1989–2004.

**ND = not determined

Several notable associations were found between ALL and AML and infant characteristics (Table 4). Male sex increased the risk for ALL (HR = 1.38, 95% CI 1.16–1.65) only. Birth weight was positively associated with both ALL and AML with HRs of 1.12 (95% CI, 1.03–1.23) and 1.33 (95% CI 1.06–1.66), respectively, for each 581 gram (1 sd) increase. When risk was examined by birth weight percentile, individuals above the 25^th ^percentile (>3117 grams) relative to those in the 5^th^-25^th ^percentile (2496–3117 grams) were at increased risk for ALL and AML. A pattern consistent with a linear relation between birth weight and ALL was observed for all percentile categories above the reference with the highest risk for individuals above the 95^th ^percentile (HR = 1.82, 95% CI, 1.22–2.73). Large size for gestational age (HR_ALL _= 1.30, 95% CI, 1.07–1.59, HR_AML _= 1.51; 95% CI, 0.95–2.40), and low one minute Apgar scores (HR_ALL _= 1.32, 95% CI, 1.08–1.61, HR_AML _= 1.62, 95% CI, 1.01–2.60) increased the risk for both leukemia subtypes. Down syndrome was associated with an increased risk for ALL of 19.8 (95% CI, 2.64–148.59) and for AML of approximately 300-fold (HR = 330, 95% CI, 94–1161) (data not shown), similar to what has been previously reported [12].

**Table 4:** Birth year adjusted analyses of the association between infant characteristics and childhood leukemia.

	**Sub-cohort**	**ALL**			**AML**		
	**No. (%)**	**No. (%)**	**HR**	**95% CI**	**No. (%)**	**HR**	**95% CI**

**Child gender**							
female	4242 (49)	227 (41)	ref.		43 (49)	ref.	
male	4501 (51)	332 (59)	1.38	1.16–1.64	44 (51)	0.97	0.64–1.49
missing	7 (0)	1 (0)			0 (0)		
**Birth weight**							
Per 1 sd increase (per 581 grams)	8710	555	1.12	1.03–1.23	87	1.33	1.06–1.66
**Birth weight category**							
< 2496 g	406 (5)	29 (5)	1.49	0.96–2.30	2 (2)	0.86	0.19–3.94
2496–3117 g	1746 (20)	83 (15)	ref.		10 (11)	ref.	
3118–3458 g	2192 (25)	117 (21)	1.13	0.84–1.51	23 (26)	1.85	0.88–3.91
3459–3798 g	2142 (25)	152 (27)	1.48	1.12–1.95	20 (23)	1.65	0.77–3.54
3799–4309 g	1796 (21)	137 (25)	1.59	1.20–2.11	27 (31)	2.65	1.27–5.49
≥ 4309 g	428 (5)	37 (7)	1.82	1.22–2.73	5 (6)	1.96	0.66–5.78
missing	40 (0)	5 (1)			0 (0)		
Size for gestational age*							
small	271 (3)	20 (4)	1.23	0.77–1.97	2 (2)	0.78	0.19–3.22
average	5708 (72)	343 (67)	ref.		54 (65)	ref.	
large	1905 (24)	151 (29)	1.30	1.07–1.59	27 (33)	1.51	0.95–2.40
missing	314 (4)	27 (5)			1 (1)		
Gestational age category*							
<37 weeks	657 (8)	43 (8)	1.00	0.72–1.38	4 (5)	0.56	0.20–1.53
37–42 weeks	6938 (88)	446 (86)	ref.		1 (1)	ref.	
>42 weeks	311 (4)	28 (5)	1.47	0.98–2.19	2 (2)	0.59	0.14–2.43
missing	292 (4)	24 (4)			1 (1)		
**One minute Apgar score**^+^							
0–7	1798 (23)	144 (29)	1.32	1.08–1.61	26 (33)	1.62	1.01–2.60
>7	5884 (77)	359 (71)	ref.		53 (67)	ref.	
missing	336 (4)	34 (6)			4 (5)		
**Five minute Apgar score**^+^							
0–7	275 (4)	14 (3)	0.77	0.44–1.33	4 (5)	1.47	0.53–4.08
>7	7394 (96)	488 (97)	ref.		75 (95)	ref.	
missing	349 (4)	35 (7)			4 (5)		
**Assisted ventilation category**^†^							
any	79 (2)	5 (2)	0.93	0.37–2.34	0 (0)	ND^‡^	
none	4548 (98)	300 (98)	ref.		41 (100)		
missing	356 (7)	31 (9)			6 (13)		
**Congenital abnormality**							
any	122 (1)	10 (2)	1.30	0.68–2.51	1 (1)	ND	
none	8100 (99)	505 (98)	ref.		81 (99)		
missing	528 (6)	45 (8)			5 (6)		

*collected 1980–2004.

^+^collected 1981–2004.

^†^collected 1989–2004.

^‡^ND = not determined

No statistically significant differences in risk between sexes were noted for any parental or infant characteristics for ALL with the exception of maternal education (p = 0.04) in birth year adjusted models, which was associated with an approximately thirty percent increased risk for males whose mothers had education beyond high school but not females (data not shown).

We conducted multivariate analyses for cases diagnosed at 0–14 years of age (Table 5) and by age strata (0–4 years, 5–14 years) (data not shown). Multivariate models included: maternal age, maternal ethnicity, last live birth interval, sex, one minute Apgar score, maternal education, birth weight percentile category and gestational age category. Risk estimates were generally similar to birth year adjusted results for cancers diagnosed 0–14 (Table 5). No statistically significant variation in risk between age groups (0–4 years vs. 5–14 years) or sex was found for variables included in multivariate models.

**Table 5:** Multivariate analysis of the association between factors recorded in birth records and acute lymphoblastic leukemia.*^+^

	**Sub-cohort**	**Cases**		
	**No. (%)**	**No. (%)**	**HR**	**95% CI**

**Maternal age (continuous/5 year increase)**	7230	481	0.97	0.88–1.08
**Maternal Ethnicity**				
black	280 (4)	8 (2)	0.43	0.21–0.88
other/unknown	340 (5)	21 (4)	0.95	0.59–1.51
white	6610 (91)	452 (94)		ref.
**Maternal Education**				
high school	2651 (37)	160 (33)		ref.
<high school	740 (10)	46 (10)	1.11	0.77–1.58
>high school	3839 (53)	275 (57)	1.18	0.95–1.47
**Last live birth interval**				
no live births	2915 (40)	183 (38)		ref.
<3 years	2783 (38)	172 (36)	1.00	0.80–1.25
≥3 years	1532 (21)	126 (26)	1.36	1.05–1.76
**Child gender**				
female	3514 (49)	187 (39)		ref.
male	3716 (51)	294 (61)	1.43	1.18–1.74
**Birth weight percentile**				
< 5th (< 2495 g)	320 (4)	25 (5)	1.37	0.80–2.34
5th-25th (2496–3117 g)	1436 (20)	71 (15)		ref.
26th-50th (3118–3458 g)	1811 (25)	105 (22)	1.17	0.85–1.60
51st-75th (3459–3798 g)	1797 (25)	130 (27)	1.37	1.00–1.86
76th-95th (3799–4309 g)	1510 (21)	115 (24)	1.40	1.02–1.92
≥95th (≥ 4309 g)	356 (5)	35 (7)	1.71	1.11–2.65
**One minute Apgar score**				
0–7	1681 (23)	138 (29)	1.30	1.05–1.61
>7	5549 (77)	343 (71)		ref.

*All variables are mutually adjusted for each other, gestational age, and birth year.

^+^Excludes 1599 individuals with missing data or who were born prior to 1980 (the earliest year gestational age was recorded in birth records).

## Discussion

The results of this study confirm previously established risk factors for ALL: male sex, white race, and higher birth weights. Further, children whose siblings were born three or more years prior were at an increased risk for ALL, while those who had low one minute Apgar scores were at an increased risk for both leukemia subtypes. Risks for ALL by age group and sex were generally similar with the exception of that for maternal education in males.

Mothers of AML cases reported being foreign born more often than those of sub-cohort members. However, there was no distinct pattern in maternal nationality suggesting that this association may be a chance finding. AML cases were also more likely to have received inadequate prenatal care. However, a biological rationale for this association is lacking leading us to conclude that random variation is a more plausible explanation.

Mothers of AML cases were also more likely to have gained ≥30 pounds during their pregnancy in contrast to another study that showed no increased risk for AML associated with maternal gestational weight gain in excess of thirty pounds [13]. It is of interest to examine maternal weight gain and risk of leukemia because of its connection with macrosomia [14] which was associated with an increased risk of AML in this study and others discussed below. Due to small numbers we could not fully address whether the association between higher birth weights and AML was mediated in part through maternal weight gain. However, within the limitations of our data, birth weight and maternal weight gain were independent risk factors in models that included both variables (data not shown).

AML risk increased linearly with maternal age in birth year adjusted analyses. Of note is that maternal age also had a significant linear effect on ALL risk in 0–4 year olds in birth year adjusted models (data not shown). The relation between advanced maternal age (defined as ≥35 in most studies) and leukemia has been examined in many studies with inconsistent findings. Studies reviewed by Little published prior to 1998 and those since for leukemia overall or ALL also support a slight positive association with a mean risk estimate of around 1.2 for mothers who were ≥35 years at the time of birth compared to younger mothers [4,13,15-25]. Most recent studies also generally support an increased risk for AML with a mean risk estimate of approximately 1.5 for mothers who were at least 35 at the time of birth vs. younger mothers [13,15,18,19,22-24]. Studies that have reported risks for ALL by age group have generally supported stronger risks for younger children associated with older maternal ages [13,15,23,25]. Confirmation of increased risk due to advanced maternal age may point to particular mechanisms, such as age associated genetic changes, in need of further study that may help to inform leukemia etiology research and reproductive decision making.

Individuals who were ≥ 3 years younger than their previous live-born sibling were at increased risk for ALL relative to children whose mothers had no other live births. Longer birth intervals have been associated with adverse maternal outcomes, particularly labor complications and pre-eclampsia [26]. In our study, the risk for ALL associated with maternal hypertension, was elevated by 30% but was consistent with chance. Pre-eclampsia and hypertension during pregnancy have been examined in at least 6 other studies with inconsistent results [4].

Substantial evidence indicates that high birth weight (≥ 4000 grams) is a risk factor for childhood leukemia, particularly ALL [5]. Our results add to this evidence and are consistent with a linear model for birth weights above 2,496 grams. The relation between high birth weight and AML is less clear but the weight of the evidence favors an association [5]. Our results also support a biological link between higher birth weights and AML and favor a linear model, within the confines of our sample size. Larger infants may be at an increased leukemia risk due to increased fetal insulin like growth factor 1 (IGF-1) exposure [27] which has been shown to be positively correlated with birth weight [28-31] and is a known mitogen for hematopoietic cells [32]. Increased levels of circulating IGF-1 may offer a proliferative advantage to initiated cells, allowing increased opportunity for malignant transformation.

Low one minute Apgar scores increased the risk for both AML and ALL. Apgar scores are used to rapidly evaluate the general health of the infant shortly after delivery with values above 7 considered excellent while values below 7 can indicate an infant who has undergone oxygen deprivation and is at increased risk of neonatal death [33]. Because infants with low Apgar scores often receive oxygen [34], a low Apgar score could indicate an infant who has undergone oxidative stress during delivery and/or who has been exposed to downstream potentially carcinogenic treatments [35]. Although oxygen exposure, as indicated by assisted ventilation, was not associated with leukemia in our study, we had inadequate power to fully address this hypothesis. At least three studies have reported positive associations between low Apgar scores and childhood leukemia or childhood cancer in general [36-38]. Two studies have reported increased risks for ALL [36] or childhood cancer overall associated with neonatal oxygen exposure [38]. Oxygen exposure after birth represents an understudied area with respect to childhood leukemia. Additional data is required to fully understand the impact of newborn medical treatments on risk.

One of the strongest and yet unexplained risk factors for childhood ALL is male sex. We did not find any statistical differences in risk factors by sex with the exception of maternal education which seems unlikely to be due to anything more than chance. Few studies have reported sex-specific risk estimates. Three studies that examined the birth weight association in sex-specific analyses have reported divergent results with one study showing that the increased risk for higher birth weights was limited to males [39] and two others showing the opposite [22,40]. In our study risk estimates were in the same direction and not statistically significant between sexes for birth weight modeled as a continuous or categorical variable. A recent review hypothesized that sex dimorphism in the risk for complex diseases may be related to differential epigenetic gene regulation by sex hormones [41]. Sex specific epigenetic differences have not been explored, to our knowledge, in the regulation of lymphoid cell lineage gene expression.

A major strength of our study is the case-cohort study design and collection of exposure data prior to disease onset obviating recall bias of particular concern in case-control studies frequently collecting data through parental interview after disease status is known. Retrospective case-control studies are also susceptible to selection bias, where estimates could be biased if the exposure distribution of the comparison population is not representative of the source cohort. Because we randomly sampled members of the sub-cohort from the entire birth cohort distribution of cases, selection bias was avoided.

Our study also has limitations. We did not have follow-up information on non-case members of the sub-cohort or have knowledge of cases diagnosed prior to 1988 (the year of inception of the cancer registry). It is also possible that some sub-cohort members who were later diagnosed with leukemia were not captured by MCSS due to out-migration. However, given the rarity of childhood leukemia, it is unlikely that out-migration or missed sub-cohort diagnoses prior to 1988 could have caused a large bias in our results. Also of note is that the success of matching cases captured by MCSS to birth records decreased with diagnosis age. The most likely reason for this is due to immigration of individuals born elsewhere which would not have biased our results because our study was of Minnesota born children. A number of individuals had missing data for variables recorded in birth records. Biased estimates from missing data occur when the data are not missing at random [42]. We do not believe that this could have caused a large bias in our results because the percentage of cases compared to sub-cohort members with missing data was similar for any given variable. Other limitations include the validity of birth record variables; variables with high validity include birth weight, Apgar scores, and delivery method. Variables that are less well measured include prenatal care, and complications of pregnancy, labor and delivery [43]. Sociodemographic variables have been generally found to be reliable in at least two studies [44,45]. Most of the findings reported here are for variables that are considered accurate and therefore most likely provide a valid estimate of risk. Finally, some of our findings may have resulted from chance variation in the distribution of exposures between cases and controls due to small numbers and multiple comparisons.

## Conclusion

We have reported findings from a population-based case-cohort study of childhood leukemia in Minnesota. Our study adds to the current knowledge of leukemia risk factors, particularly birth weight with evidence supporting a positive linear relation between birth weight and both ALL and AML. Our data also suggests that early-life factors recorded in birth records do not explain the male excess found in ALL. Further, we have shown an increased risk between low Apgar scores and leukemia, the reasons for which should be the subject of further investigation.

## Competing interests

The author(s) declare that they have no competing interests.

## Authors' contributions

KJ conducted the analysis and prepared the paper for editing by coauthors and submission. JS assembled the data for analyses, retrieved additional data elements from the cancer and birth registries, and reviewed and edited the manuscript. SP provided statistical assistance and reviewed and edited the manuscript. JR & LS reviewed the proposal for the manuscript, all analyses, and edited manuscript drafts.

## Pre-publication history

The pre-publication history for this paper can be accessed here:


